# Trabectedin in Soft Tissue Sarcomas

**DOI:** 10.3390/md13020974

**Published:** 2015-02-12

**Authors:** Bradley J. Petek, Elizabeth T. Loggers, Seth M. Pollack, Robin L. Jones

**Affiliations:** 1 University of Washington, 1959 NE Pacific St, Seattle, WA 98195, USA; E-Mail: bpetek@uw.edu; 2 Fred Hutchinson Cancer Research Center, University of Washington, 825 Eastlake Avenue East, G-3630, Seattle, WA 98109-1023, USA; E-Mails: eloggers@seattlecca.org (E.T.L.); spollack@fhcrc.org (S.M.P.)

**Keywords:** trabectedin, ET-743, DNA minor groove binder, soft tissue sarcoma, chemotherapy

## Abstract

Soft tissue sarcomas are a group of rare tumors derived from mesenchymal tissue, accounting for about 1% of adult cancers. There are over 60 different histological subtypes, each with their own unique biological behavior and response to systemic therapy. The outcome for patients with metastatic soft tissue sarcoma is poor with few available systemic treatment options. For decades, the mainstay of management has consisted of doxorubicin with or without ifosfamide. Trabectedin is a synthetic agent derived from the Caribbean tunicate, *Ecteinascidia turbinata*. This drug has a number of potential mechanisms of action, including binding the DNA minor groove, interfering with DNA repair pathways and the cell cycle, as well as interacting with transcription factors. Several phase II trials have shown that trabectedin has activity in anthracycline and alkylating agent-resistant soft tissue sarcoma and suggest use in the second- and third-line setting. More recently, trabectedin has shown similar progression-free survival to doxorubicin in the first-line setting and significant activity in liposarcoma and leiomyosarcoma subtypes. Trabectedin has shown a favorable toxicity profile and has been approved in over 70 countries for the treatment of metastatic soft tissue sarcoma. This manuscript will review the development of trabectedin in soft tissue sarcomas.

## 1. Introduction

Soft tissue sarcomas are a group of rare solid tumors of mesenchymal origin. Surgical resection with or without radiation is the mainstay of management for localized disease. However, approximately 50% of patients with high-grade tumors will develop recurrent disease. Systemic therapy may additionally be considered in the localized setting, but its role in management remains controversial. The outcome of patients with metastatic soft tissue sarcoma is poor with a median overall survival (OS) of about 12 months. Systemic therapy is at the core of management for patients with metastatic disease; however, there are few effective agents available. Doxorubicin and ifosfamide have shown consistent activity, and recently, the anti-angiogenic agent, pazopanib, has been approved based on the results of a randomized, placebo-controlled phase III trial showing a significant, but modest, benefit in progression-free survival (PFS) for patients treated with pazopanib [1].

Trabectedin is a synthetic, marine-derived alkylating agent derived from the Caribbean tunicate, *Ecteinascidia turbinata* [2]. The success of trabectedin in preliminary clinical trials for soft tissue sarcomas has led to the approval of the drug in many countries and a large, randomized phase III trial. With limited systemic therapy options available for sarcomas as a whole, trabectedin has the opportunity to be significantly beneficial for patients with metastatic disease.

## 2. Mechanism of Action

**Figure 1:**
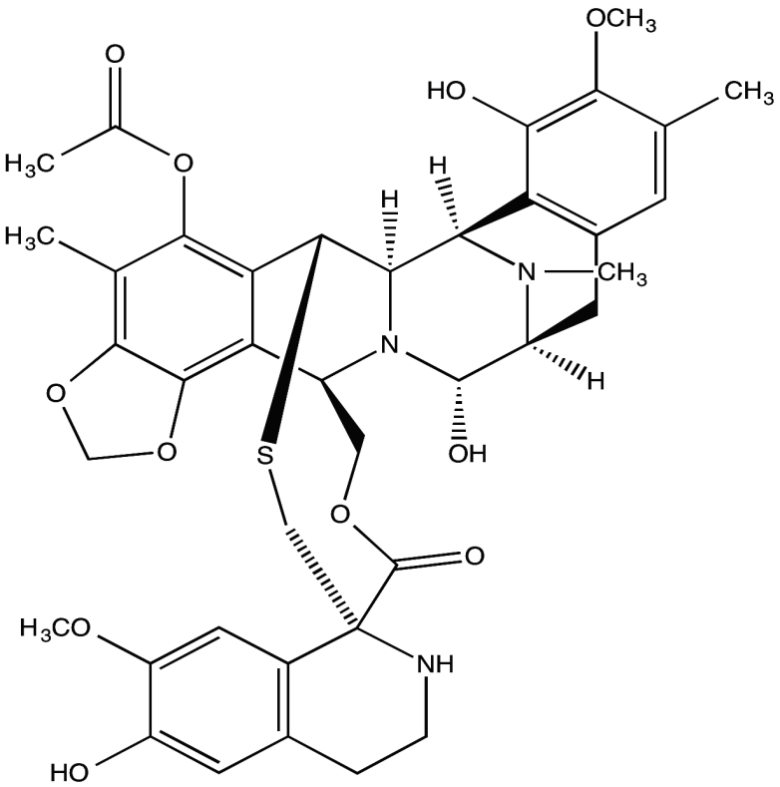
Chemical structure of trabectedin.

Trabectedin is a natural alkaloid composed of three tetrahydroisoquinolone rings (Figure 1). The unique structure of trabectedin allows it to bind to the N2 amino group of guanine residues in the minor groove of the DNA double helix and can lead to double-strand breaks [3,4]. Studies have suggested that the nucleotide excision repair mechanism may be essential for the antitumor activity of trabectedin and that the resulting double-strand breaks are more persistent if there is loss of homologous repair [5,6]. Evidence shows that disruption of DNA by trabectedin ultimately causes apoptosis and sensitization of cancer cells to Fas-mediated cell death [7]. Multiple studies, including specific experiments in soft tissue sarcomas, have also validated that trabectedin works at the tumor microenvironment with selective activity against monocytes and tumor-associated macrophages [8,9]. The inhibition of these immune factors allows for the prevention of angiogenesis and further disease progression [10,11]. Deprivation of inflammatory-mediated support in the tumor microenvironment may be one of the most important aspects of trabectedin’s mechanism of action, making the drug efficacious as a cancer treatment. Other mechanisms for the chemotherapeutic actions of trabectedin may include modulation of the cell cycle and interaction with transcription factors [12,13].

Recent studies have also proposed specific mechanisms of action for trabectedin in myxoid/round cell liposarcoma. The most common translocation in the disease is t(12;16)(q13;p11), creating a *FUS-CHOP* fusion gene, and rarely, a t(12;22)(q13;q12) translocation takes place resulting in a *EWS-CHOP* fusion gene. These fusion-encoded proteins are believed to function as abnormal transcription factors [14]. Notably, an* in vivo* study showed that mesenchymal stem cells expressing the FUS-CHOP protein were committed to adipocytic differentiation, but were unable to terminally differentiate [15]. Trabectedin administration in this experiment downregulated *FUS:CHOP* expression, which promoted terminal adipocytic differentiation. Others have hypothesized that trabectedin prevents the binding of the FUS-CHOP oncogenic chimera protein to its target promoters, which may modulate the transcription of genes that are essential for adipocytic differentiation [14,16]. Recent studies have also characterized regulatory networks leading to trabectedin resistance, as well as have uncovered the antiangiogenic activity of trabectedin in myxoid/round cell liposarcoma [17,18].

## 3. Phase I Trials

A number of phase I trials have assessed differential dosing and infusion schedules for the administration of trabectedin [19,20,21,22,23,24,25]. Results from these studies have established the optimal regimen of administration to be a 1.5-mg/m^2^ infusion over 24 h every three weeks [26]. Notably, Taamma and colleagues performed a phase I trial of trabectedin in 52 patients with treatment refractory tumors that recommended the current optimal dosing schedule of 1.5 mg/m^2^ for a 24-h continuous infusion [22]. The most prevalent dose limiting toxicities in the study were hematological in nature. At the recommended dose, severe neutropenia was reported in 33% of cycles, severe thrombocytopenia in 10% of cycles and reversible, but severe, elevations in transaminase levels in 38% of cycles. The investigators also observed that patients with baseline hepatobiliary function abnormalities had a higher likelihood of severe hematological toxicity, indicating the need for dose reduction in such patients.

Combination therapies of trabectedin with other chemotherapeutic agents, including gemcitabine, doxorubicin, Doxil and cisplatin, have also been assessed in phase I trials [27,28,29,30,31,32]. The most promising results from these studies in sarcoma patients have been from the trials administering trabectedin in combination with doxorubicin. One of the trials showed a response rate (RR) of 18% and stable disease in 56% of soft tissue sarcoma patients (*n* = 29) [31]. Another study assessing combination therapy with doxorubicin reported a RR of 12% with median PFS of 9.2 months for 41 patients with solid tumors [27]. Of the 41 patients, 20 had liposarcoma and 11 had leiomyosarcoma. With success in phase I trials, the efficacy of combination therapy with doxorubicin and trabectedin is currently under investigation in larger studies.

## 4. Phase II Trials

Two phase II trials in 2004 provided the initial analysis of trabectedin in soft tissue sarcomas. The first of these studies was run on 54 pretreated soft tissue sarcoma patients and reported a low response rate of 4%, but a high disease control rate at six months of 24% [33]. Trabectedin was administered at a dose of 1.5 mg/m^2^, over 24 h every three weeks. Approximately half of the patients in the study eventually developed grade 3/4 aspartate aminotransferase (AST) and alanine aminotransferase (ALT) levels. Another common adverse event was neutropenia, being grade 3/4 in 61% of patients. In this trial, four patients (7.4%) discontinued trabectedin due to adverse events. In addition, there were two treatment-related deaths, and both were patients who developed acute renal failure. 

The second phase II trial, published in 2004, again reported a low response rate of 8% and a one-year overall survival rate of 53% in 36 previously treated sarcoma patients [34]. This study also utilized the same dosing schedule of trabectedin (1.5 mg/m^2^, over 24 h every three weeks). The toxicity profile of the drug was similar to previous trials with patients experiencing elevations in transaminases, fatigue and hematological toxicity. Growth factors, such as, granulocyte-colony stimulating factor (G-CSF), can be administered to help prevent hematological toxicity; however, a recent retrospective study showed that G-CSF administration has only been used in about 10% of phase II trials on trabectedin in solid tumors [35].

Early promising results in these phase II studies led the European Organization for the Research and Treatment of Cancer (EORTC) to initiate a phase II trial of trabectedin in 104 patients treated in the second- and third-line setting [36]. This trial also reported a low response rate of 8%. The six-month PFS was 29%, and the median overall survival (OS) was reported as 9.2 months. Subsequently, a further phase II trial in 36 patients was run to evaluate the activity of trabectedin in the first-line setting. The overall response rate was reported as 17%, and the one-year PFS and OS rates were 21% and 72%, respectively [37]. This study also importantly concluded that trabectedin has similar ranges of objective responses and overall survival rates in the first-line setting to the two most active drugs in soft tissue sarcomas: doxorubicin and ifosfamide.

Demetri and colleagues performed a phase II trial randomizing 270 patients with leiomyosarcoma and liposarcoma to receive either 1.5 mg/m^2^ of trabectedin over 24 h every three weeks, or 0.58 mg/m^2^ over 3 h every week for three out of four weeks [38]. Patients were required to have experienced documented disease progression while on doxorubicin and ifosfamide prior to trial entry. The 24-h infusion schedule showed a significantly longer median time to progression (TTP) (3.7* vs.* 2.3 months) and PFS (3.3* vs.* 2.3 months), as compared to the 3-h infusion schedule. No significant difference in overall survival was observed between the two arms of the trial; however, there was a strong trend favoring the 24-h infusion schedule (13.9 months* vs.* 11.8 months). Trabectedin was generally well tolerated in this study, with the most frequently reported grade 3/4 adverse events being neutropenia and elevated transaminases. Febrile neutropenia occurred in 1% of patients, and the majority of adverse events were mild to moderate. There was also no documentation of cumulative toxicities. Another phase II trial recommended the use of trabectedin as a neoadjuvant therapy for patients with advanced myxoid liposarcoma [39].

The results of these phase II trials led to licensing approval of trabectedin by the European Union for advanced soft tissue sarcoma in 2007, and the drug is now approved in over 70 countries.

## 5. Phase III Trials

Trabectedin has yet to be approved by the FDA in the United States, and consequently a phase III trial has been performed randomizing over 500 patients with leiomyosarcoma and liposarcoma to receive either trabectedin or dacarbazine (2:1 ratio). The primary end point of the trial is overall survival. This trial has closed to enrollment, and the results are eagerly awaited (NCT01343277).

In addition, there is an ongoing worldwide, expanded access program (NCT00210665). A recent analysis from this study reported that of the 1,895 soft tissue sarcoma patients entered into the program, patients with leiomyosarcoma and liposarcoma had significantly longer OS compared to all other histological subtypes (16.2* vs.* 8.4 months, respectively), as well as a higher objective response rate (6.9%* vs.* 4%, respectively) [40]. 

Another phase III trial randomized 121 patients with translocation-related sarcomas to receive trabectedin or doxorubicin in the first-line setting with progression-free survival as the primary end point [41]. There was no significant difference in PFS between the two arms of the trial. At the time of analysis, 63.9% and 58.3% of patients were alive in the trabectedin and doxorubicin arms, respectively (with no statistically significant difference in overall survival). The response rate according to RECIST 1.0 was significantly higher in the doxorubicin (27%) compared to the trabectedin (5.9%) arm of the trial. In contrast, the response according to Choi criteria showed fewer differences between the doxorubicin (45.9%) and trabectedin (37.3%) arms, in terms of response rate. 

## 6. Retrospective Studies

Trabectedin has shown particular activity in myxoid/round cell liposarcoma. In a retrospective analysis of 51 patients treated at five referral centers, a median overall response rate of 51% was reported, and the median PFS was 14 months [42]. Progression-free survival at six months was also reported as 88%. In another retrospective study assessing the efficacy of trabectedin in specific translocation-related sarcomas, the rate of PFS at six months was documented as 64% in myxoid/round cell liposarcoma (*n* = 27) and 22% in synovial sarcoma (*n* = 45) [43]. These results suggest that trabectedin may have significant activity in these two histological subtypes and should be further analyzed in larger randomized clinical trials.

Other retrospective studies have shown that trabectedin may also be effective in uterine leiomyosarcoma. In a study assessing 66 patients with metastatic uterine leiomyosarcoma, PFS for the entire cohort was 3.3 months with 16% of patients achieving a partial response and 35% showing stable disease [44]. Furthermore, a prospective phase II trial of trabectedin in uterine leiomyosarcoma reported a median PFS of 5.8 months and OS of 26.1 months [45]. Two patients in the study also had a partial response to trabectedin administration (10%).

Retrospective analysis was also used to assess the effect of age on the efficacy and safety of trabectedin administration in the treatment of soft tissue sarcomas [46]. Patients under the age of 60 were part of the younger cohort, and patients 60 or older were a part of the older cohort. The analysis pooled five prior phase II trials and showed similar response rates (younger 10.1%* vs.* older 9.6%), no significant difference in median progression-free survival (younger 2.5* vs.* older 3.7 months) and similar overall survival rates between cohorts (younger 13.0* vs.* older 14.0 months). However, older patients did show a higher incidence of grade 3/4 neutropenia (43.6%* vs.* 60.2%) and fatigue (6.3%* vs.* 14.4%). A small subset of patients 70 or older were also included in the analysis and showed no significant differences in efficacy or safety outcomes. This study therefore indicates that trabectedin can have meaningful benefits and an acceptable safety profile in young and elderly patients.

## 7. Conclusions

Trabectedin has shown consistent activity in patients with previously treated soft tissue sarcoma. The results of a number of phase II trials have led to the drug being approved in over 70 countries worldwide. The results of a phase III trial randomizing leiomyosarcoma and liposarcoma patients to receive trabectedin or dacarbazine are awaited and could lead to the drug being approved by the FDA.

Further evaluation is required for combination strategies of trabectedin, particularly with doxorubicin [27,31]. Investigation of this agent in the neoadjuvant and adjuvant setting may also be warranted in certain histological subtypes. 
